# Changes in free amino acids and hardness in round of Okinawan delivered cow beef during dry- and wet-aging processes

**DOI:** 10.1186/s40781-018-0180-x

**Published:** 2018-09-25

**Authors:** Takashi Hanagasaki, Naokazu Asato

**Affiliations:** 1 Okinawa prefectural Industrial Technology Center, 12-2 Suzaki, Uruma-shi, Okinawa 904-2234 Japan; 2 Okinawa prefectural Livestock Research Center, 2009-5 Shoshi, Nakizin-son, Okinawa 905-0426 Japan; 3 Present address: Okinawa prefectural Agricultural Research center, 820 Makabe, Itoman city, Okinawa 901-0336 Japan; 4 Present address: Livestock Division of Okinawa prefectural government, 1–2-2 Izumizaki, Naha city, Okinawa 900–8570 Japan

**Keywords:** Dry aging beef, Okinawan delivered cow, Free amino acids and hardness

## Abstract

**Background:**

Aging trials are conducted to determine characteristics associated with dry- and wet-aging processes of beef from delivered cows grown in Okinawa, i.e., dams that have finished giving birth (Okinawan delivered cow beef). Changes in free amino acids, hardness, and other factors were analyzed in round of Okinawan delivered cow beef during dry- and wet-aging processes along with a comparison with characteristics of beef imported from Australia.

**Results:**

Functional amino acids did not increase during both dry- and wet-aging processes. However, proteinogenic amino acids increased significantly (*P* < 0.05) and hardness tended to decrease during both dry- and wet-aging processes. On comparison between dry- and wet-aging processes by analysis of variance, drip and cooking losses were significantly lower during the dry-aging process than during the wet-aging process. However, there was no significant difference in free amino acids or hardness in this comparison.

**Conclusion:**

There was no significant difference between dry- and wet-aging methods for all studied variables related to free amino acids or hardness in this study.

## Background

The livestock industry is a major part of the Okinawan economy, with the Prefecture selling the fourth highest number of calves in Japan. In Okinawa, there are numerous dams, some of which have finished giving birth and thus, are termed as Okinawan delivered cows. The turnover for Okinawan delivered cows is approximately 5000 heads per year; these slaughtered cows are generally sold at a lower price than fattened cattle and heifers. The reason for them being less valuable is their less tender meat. However, there is significant potential for adapting these cows’ meat and increasing their value.

Dry aging is one aging method that is said to improve meat with respect to some characteristics. Recently, increased interest in dry-aged beef has emerged not only in Western countries but also in Asian countries, such as South Korea, Japan, Singapore, Taiwan, and Hong Kong [3].

Over 2.5 million foreign tourists visited Okinawa in 2017, which is the highest number recording in any year, according to the official figures of the Okinawa prefectural government. Tourists from Asian countries, particularly those from Taiwan, Hong Kong, South Korea, and China, accounted for approximately 90% of this number. In this context, there is an urgent need to boost the production and awareness of Okinawa’s unique food brands. Thus, we hope to create an Okinawan brand of dry-aged beef by adding value to Okinawan beef because it is produced in Okinawa and has high potential to become very popular in view of the region’s tourist industry.

During the dry-aging process, juices are absorbed into the meat and chemical breakdown of protein occurs, giving a more intense nutty and beefy flavor [3]. Moreover, during aging, the endogenous enzymes break down myofibrillar proteins in the muscle, which leads to more tender beef [1, 2]. Because of this, it is considered that lean meat with low fat content is more suitable for dry aging and has higher potential to undergo a change in its quality. Specifically, round of beef (i.e., from the rear leg of the cow) is most likely to be appropriate for dry aging. Indeed, dry-aging products using round of beef have already been commercialized in Okinawa.

In general, the dry-aging process is performed under aerated conditions, whereas the wet-aging process essentially involves vacuuming and packaging, which means that the conditions do not involve aeration. Savell [7] reported a survey on consumer preferences between dry-aged and wet-aged beef in an executive summary of the National Cattlemen’s Beef Association’s Center in the United States of America (hereinafter referred to as “USMEF”). There is only a little thing known about specific differences by scientific approach between beef aged by these two methods. Warren and Kastner [11] obtained the result that dry-aged steaks had significantly higher beefy and brown/roasted flavor intensities than the unaged or vacuum-aged steaks, whereas vacuum-aged steaks had significantly higher bloody/serumy and sour flavor intensities than the unaged or dry-aged steaks. King et al. [4] stated wet-aged beef had significantly greater percentages of acids than dry-aged beef. In terms of hardness, Parrish Jr. et al. [6] found that rib and loin steaks from their wet aging treatment were significantly more tender than the rib and loin steaks from their dry aging treatment. However, Warren and Kastner [11] found that both vacuum aging and dry aging for 11 days resulted in tenderness scores that were significantly higher than the unaged controls. To shed light on this issue, in this study, scientific data, such as free amino acids and hardness, were analyzed in the round of Okinawan delivered cow beef during dry-aging and wet-aging processes along with a comparison with beef imported from Australia (hereinafter referred to as Australian beef).

## Methods

### Animals and muscle samples

Two types of beef were used for the aging experiments: one from Okinawan delivered cows that were > 10 years old and thus, had already finished giving birth and the other one Australian beef. Type of Okinawan delivered cow beef was a kind of Japanese cow called black-haired Japanese cow. Type of Australian beef was crossbreed thought to be in the family lineage of black cattle mainly, which was raised in pastures. With regard to Okinawan delivered cow beef, two chunks of meat (approximately 6 kg/each) from the round in the same position of both right and left sides of the carcass from one individual were used for both dry-aging and wet-aging experiments. The same approach was also applied to two other individuals, so there were three pieces for each aging experiment. With regard to Australian beef, six chunks of meat (approximately 6 kg/each) from the round of each individual were grouped into dry-aging and wet-aging experiments, namely, three in each group. Each chunk of meat was divided into five pieces by cutting for experiments involving 0, 1, 2, 3, or 4 weeks of aging.

### Methods

#### Aging environment

The dry-aging environment was created in a refrigerator (Showa Denko K.K., Tokyo, Japan) in Okinawa Industrial Technology Center at a temperature of 2 °C. Dry boxes were placed in the refrigerator for the dry-aging experiment. Three pieces of meat were put in the dry box without a fan for each week under maintained conditions of approximately 80% relative humidity and no air flow. The pieces of meat for the wet-aging experiment, which were vacuumed and packaged, were placed in a refrigerator set at 2 °C during the aging process, the same as that used for the dry-aging experiment.

#### Measurement of moisture and trim losses

Moisture loss is the weight of water lost from meat and is determined by measuring the difference in meat weight between before and after it has been subjected to dry aging. Trim loss is weight of the trimming part of meat that is discolored and dehydrated. Productive loss is the sum of moisture and trim losses.

Samples for these measurements were cut to a size of approximately 2.5 cm (length) × 2.5 cm (width) × 1 cm (height) at a depth of over 1 cm from the surface of the meat and were frozen until subsequent analyses. Prior to analyses, these frozen samples were kept at a normal temperature for 2.5 h and weighed before their drip was removed. The weight of these samples was measured again after their drip had been removed. The weight difference was calculated and was denoted as a ratio relative to the initial weight. The percentage of drip loss was calculated in this way. In terms of cooking loss, samples that had already undergone drip loss measurement were put into a plastic bag and incubated at 70°C in a water bath for 1 h. After they had been cooled and their drip had been removed, the weight of these samples was measured. The weight difference between before and after incubation was determined as a ratio relative to the weight before incubation [5].

#### Quantitative analysis of amino acids

First, sampling for the dry-aging experiment for 2, 3, and 4 weeks was performed to a depth of over 1 cm from the surface of the edible part, after it had been trimmed. The sampling for the dry-aging experiment for 0 and 1 week and the wet-aging experiment for 0, 1, 2, 3, and 4 weeks was performed to a depth of over 1 cm from the surface of the meat without trimming. These were cut and homogenized. Extract solutions were obtained from these homogenized samples after protein was removed with acetonitrile and perchlorate and fat was removed with hexane. Sample solutions for LC/MS were prepared after extract solutions had been filtered. Sample solutions were injected onto an Intrada Amino Acid column (3 × 100 mm, Imtakt Corp., Kyoto, Japan) at a flow rate of 0.6 ml/min. The separation was performed with a two-pump gradient. Solvent A was acetonitrile/tetrahydrofuran/25 mM ammonium formate/formic acid (9/75/16/0.3, *v*/v/*v*/v). Solvent B was acetonitrile/100 mM ammonium formate (20/80, v/v). The gradient program was as follows: 0, A 100%; 2.75, A 100%; 7.75, A 83%; and 7.76 min, A 0%. Analyses were monitored in the positive-ion mode using an ESI source at 350 °C and MRM.

Amino acids were sorted according to their features into four groups. Glycine, alanine, threonine, serine, and proline were classified as sweet-tasting amino acids. Aspartic acid, glutamic acid, glutamine, and asparagine were classified as umami-tasting amino acids. Methionine, lysine, isoleucine, leucine, phenylalanine, tyrosine, valin, histidine, arginine, and cystine were classified as savory-tasting amino acids. Finally, carnosine, anserine, taurine, ornithine, and GABA were classified as functional amino acids.

### Rheological properties

Breaking stress, shearing stress, and other rheological properties of each meat sample were measured using a rheometer RE-3305S (Yamaden Co. Ltd., Tokyo, Japan) and a breaking strength analyzer BAS-33005-16 (Yamaden Co. Ltd., Tokyo, Japan). Samples for these measurements were those that had already undergone both drip and cooking loss measurements as described above. With regard to the measurement of breaking stress, this was performed on a sample of approximately 1 cm in height using a rheometer with a plunger No. 5 stick-type at a speed of 1 mm/s. Approximately seven runs were performed for each measurement and the average was calculated. The measurement of shearing stress was performed in an almost similar manner. Specifically, it was performed on samples of a size of approximately 1 cm (width) × 1 cm (height) using a rheometer with a plunger No. 21 knife-type at a speed of 1 mm/s. These samples were cut vertically on muscle fiber. Approximately five runs were performed for each measurement and the average was calculated.

### Statistical analysis

Two-way analysis of variance (ANOVA) was used for statistical analysis with the program JMP® 13 (SAS Institute Inc., Cary, NC, USA). Tukey’s test was used for identifying differences (*P* < 0.05) between in the same beef sample during the same aging process.

## Results

**Fig. 1:**
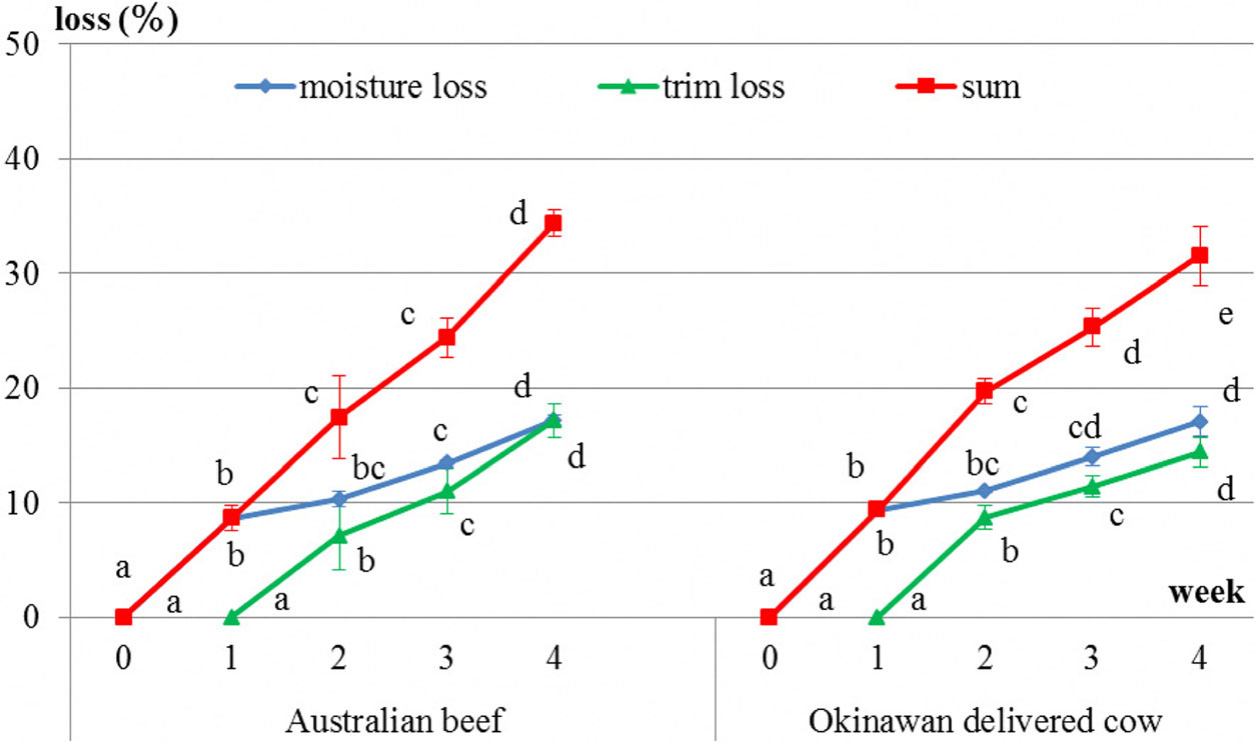
Changes in moisture and trim losses during the dry-aging process. Different letters in the same group indicate significant differences

**Fig. 2:**
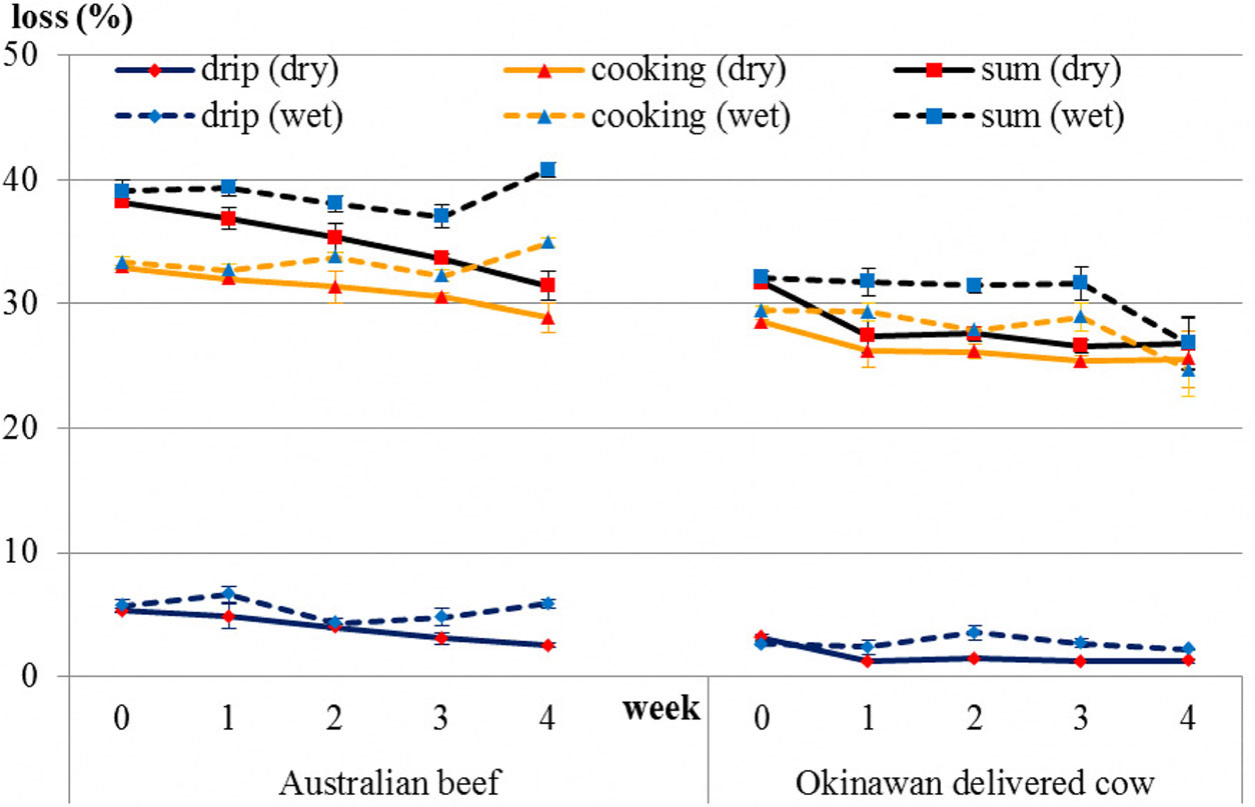
Changes in drip and cooking losses during both dry- and wet-aging processes

It was thought that moisture loss occurred immediately after the initiation of the dry-aging process, whereas trim loss occurred approximately 10 days after the dry-aging process had started. Levels of both losses increased in both beef types as the number of days of dry aging increased (Fig. 1). In Australian beef, drip loss significantly decreased and cooking loss (*P* = 0.086) showed a decreasing trend during the dry-aging process. In addition, drip loss (*P* = 0.07) showed a decreasing trend and cooking loss significantly increased during the wet-aging process. In Okinawan delivered cow beef, drip loss significantly decreased and cooking loss (*P* = 0.42) appeared to remain unchanged during the dry-aging process (Fig. 2).

**Fig. 3:**
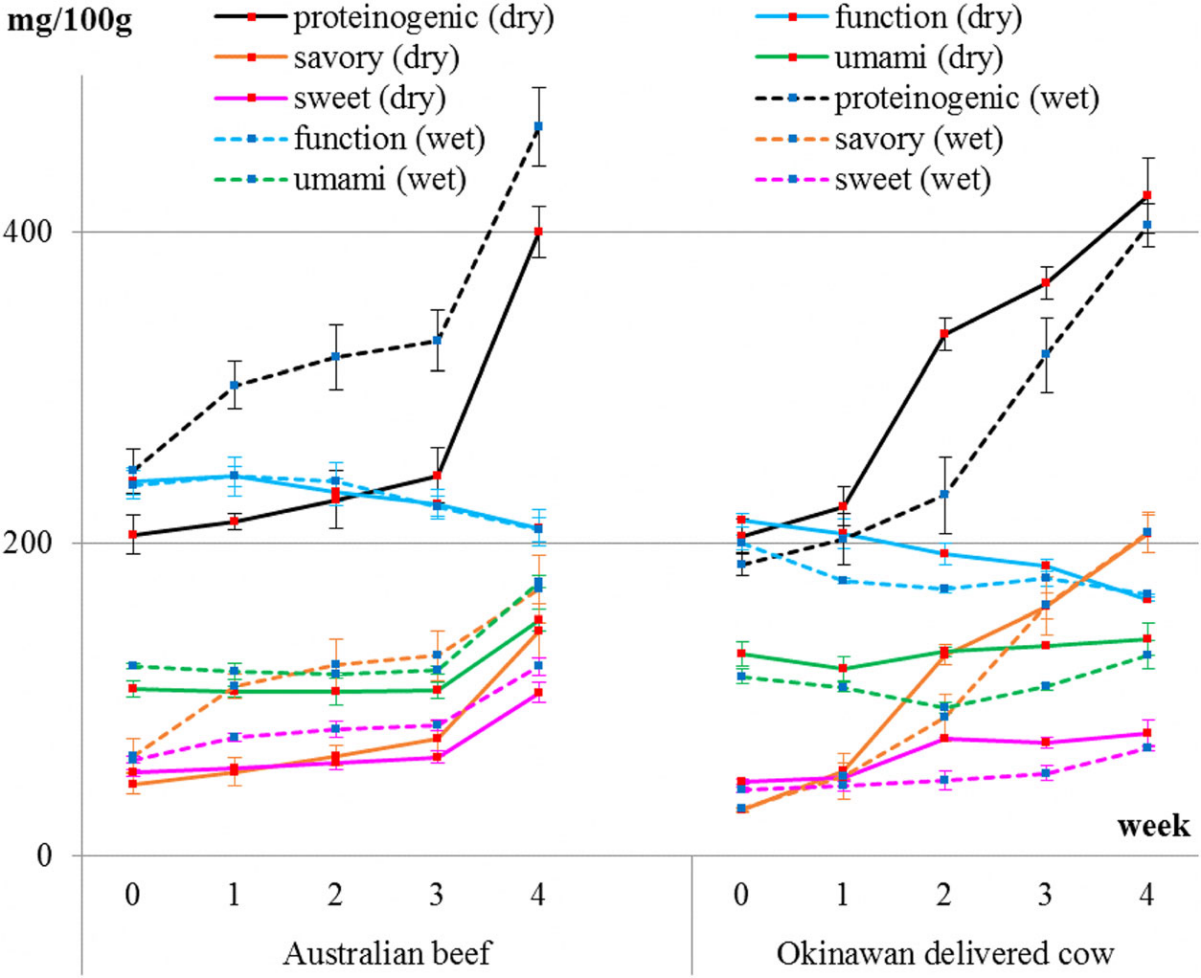
Change in levels of each amino acid group in both types of beef during both dry- and wet-aging processes. Amino acid groups are classified as sweet-, umami-, and savory-tasting and functional. The sum of sweet-, umami-, and savory-tasting amino acids constitute the proteinogenic category. Changes in their levels during the dry-aging process are shown by solid lines. Changes in their levels during the wet-aging process are shown by dotted lines

**Fig. 4:**
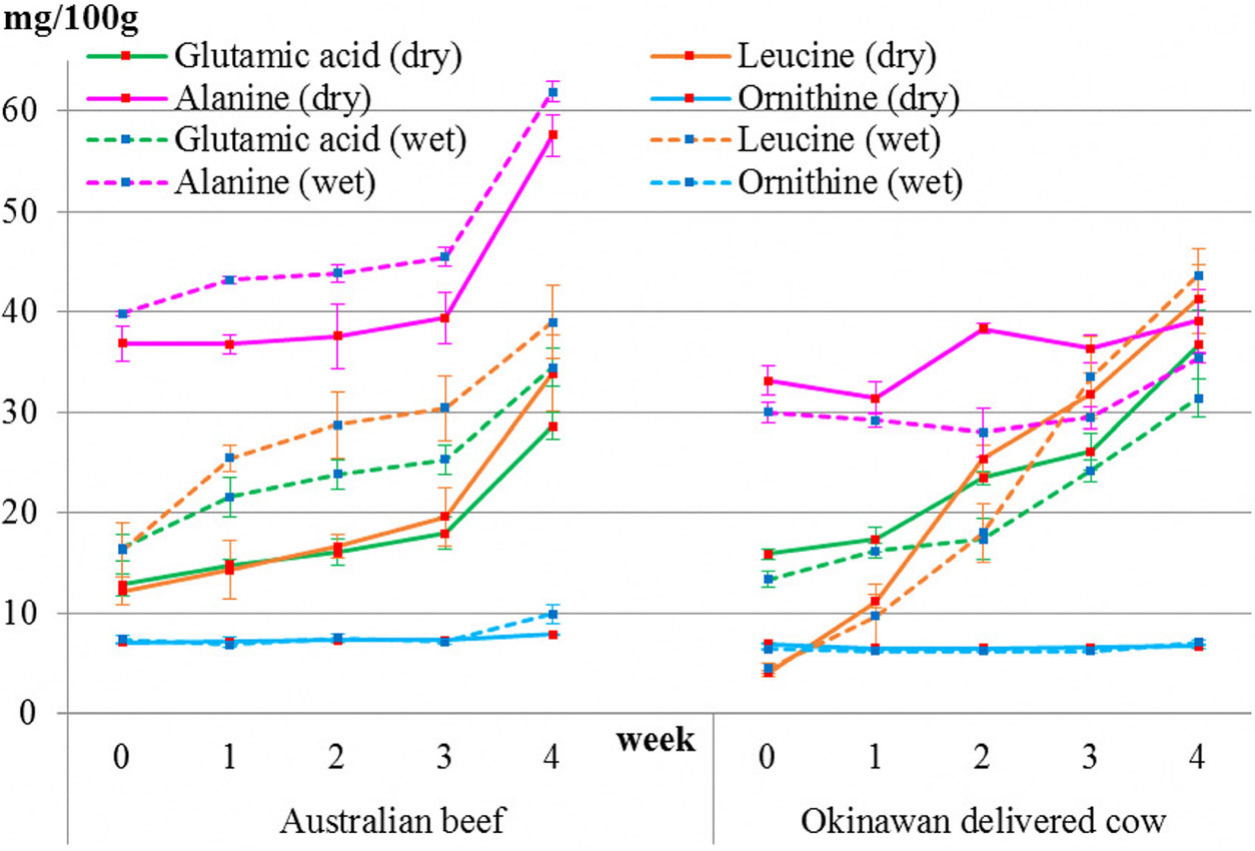
Changes in levels of alanine, glutamic acid, leucine and ornithine in both types of beef during both dry- and wet-aging processes. Changes in their levels during the dry-aging process are shown by solid lines. Those during the wet-aging process are shown by dotted lines

The results also showed that sweet-, savory-, and umami-tasting amino acids increased in both beef types during dry- and wet-aging processes (Fig. 3 and Table 1). Their sum reflects the level of proteinogenic amino acids, which also increased. However, levels of functional amino acids did not increase in both beef types during dry- and wet-aging processes, but rather decreased in Okinawan delivered cow beef. Total amino acids, including functional amino acids, in both beef types significantly increased during dry- and wet-aging processes (data not shown); this increase was almost the same as that observed for proteinogenic amino acids. GABA and cystine were not detected at all in all samples. Representative sweet-, savory-, and umami-tasting amino acids, namely, alanine, leucine, and glutamic acid, respectively, are shown (Fig. 4 and Table 2). Levels of leucine and glutamic acid increased stably in both beef types during both aging processes. In terms of alanine, it showed no increase in Okinawan delivered cow beef after dry- and wet-aging processes. In terms of ornithine, it was initially at a low level and remained constant in both beef types during both aging processes.

**Table 1:** Results of the average value ± standard deviation and Tukey’s test for each amino acid group listed in Fig. 3

Australian beef											
	week	0		1		2		3		4	
proteinogenic	dry	206±22	a	214±9	a	228±32	a	243±31	a	399±29	b
	wet	246±24	a	301±26	a	319±36	a	330±34	a	467±44	b
function	dry	239±15	a	243±22	a	233±15	a	225±16	a	210±21	a
	wet	238±16	a	243±11	a	239±22	a	224±11	a	209±14	a
umami	dry	107±9	a	105±6	a	105±15	a	106±9	a	151±12	b
	wet	121±3	a	118±9	a	116±1	a	119±5	a	175±6	b
sweet	dry	53±4	a	56±1	a	59±7	a	63±7	a	104±11	b
	wet	61±4	a	76±5	ab	81±9	b	84±6	b	121±10	c
savory	dry	46±10	a	53±16	a	64±11	a	75±20	a	144±30	b
	wet	64±19	a	108±14	ab	122±28	ab	128±28	ab	170±37	b
Okinawan delivered cow											
	week	0		1		2		3		4	
proteinogenic	dry	205±19	a	223±22	a	334±18	b	367±18	bc	423±42	c
	wet	186±12	a	202±28	a	230±43	a	321±41	b	404±24	b
function	dry	215±7	a	206±16	ab	193±12	ab	186±6	bc	164±2	c
	wet	200±8	a	176±3	b	170±4	b	178±10	b	167±0	b
umami	dry	129±13	b	120±14	a	131±5	a	135±2	a	139±18	a
	wet	115±8	ab	108±5	ab	95±6	a	108±4	ab	129±16	b
sweet	dry	47±3	a	50±4	a	75±2	b	72±6	b	78±15	b
	wet	42±3	a	44±6	a	48±11	a	53±9	ab	69±3	b
savory	dry	29±2	a	54±9	a	128±12	b	160±14	b	206±21	c
	wet	29±2	a	50±25	a	88±26	a	160±32	b	207±22	b

Different letters in the same group represent significant differences (*P* < 0.05)

**Table 2:** Results of the average value ± standard deviation and Tukey’s test for each amino acid listed in Fig. 4

Australian beef											
	week	0		1		2		3		4	
Alanine	dry	37±3	a	37±2	a	38±6	a	40±4	a	58±4	b
	wet	40±0	a	43±1	ab	44±2	b	45±2	b	62±2	c
Glutamic acid	dry	13±2	a	15±1	ab	16±2	ab	18±3	b	29±2	c
	wet	16±2	a	22±3	ab	24±3	ab	25±3	b	34±3	b
Leucine	dry	12±2	a	14±5	a	17±2	a	20±5	a	34±7	b
	wet	16±5	a	25±2	ab	29±6	ab	30±6	b	39±6	b
Ornithine	dry	7±0	a	7±1	a	7±0	a	7±0	a	8±0	a
	wet	7±1	ab	7±0	a	7±1	ab	7±1	a	10±2	b
Okinawan delivered cow											
	week	0		1		2		3		4	
Alanine	dry	33±2	ab	32±3	a	38±1	ab	36±2	ab	40±4	b
	wet	30±2	ab	29±1	ab	28±4	a	29±2	ab	35±1	b
Glutamic acid	dry	16±1	a	17±2	a	24±1	ab	26±3	b	38±5	c
	wet	13±1	a	16±1	a	17±4	a	24±2	b	31±3	c
Leucine	dry	4±1	a	11±1	a	25±2	b	32±4	b	42±5	c
	wet	5±1	a	10±5	ab	18±5	b	33±7	c	44±5	c
Ornithine	dry	7±0	a	7±0	a	7±0	a	7±0	a	7±0	a
	wet	6±0	ab	6±0	a	6±0	a	6±0	a	7±1	b

Different letters in the same group represent significant differences (*P* < 0.05)

**Fig. 5:**
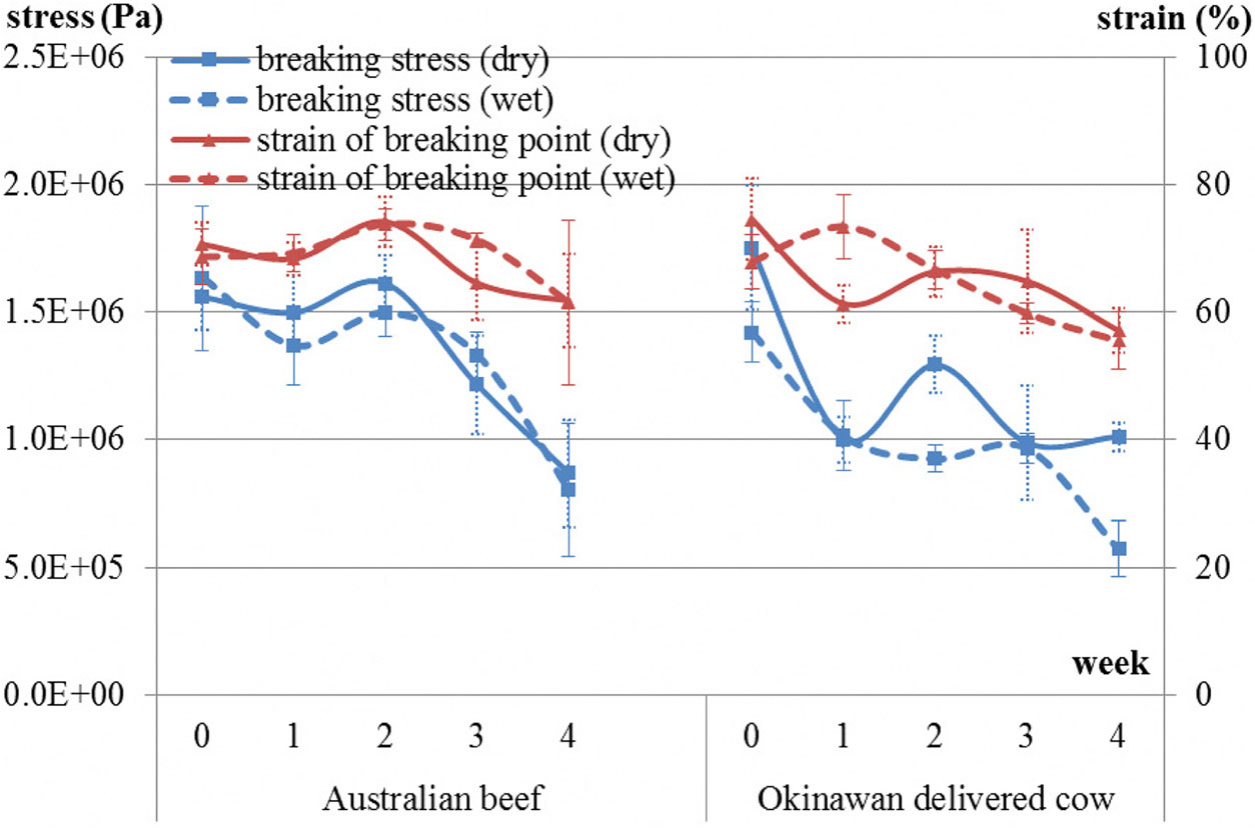
Changes in breaking stress and strain of breaking point in both types of beef during both dry- and wet-aging processes

**Fig. 6:**
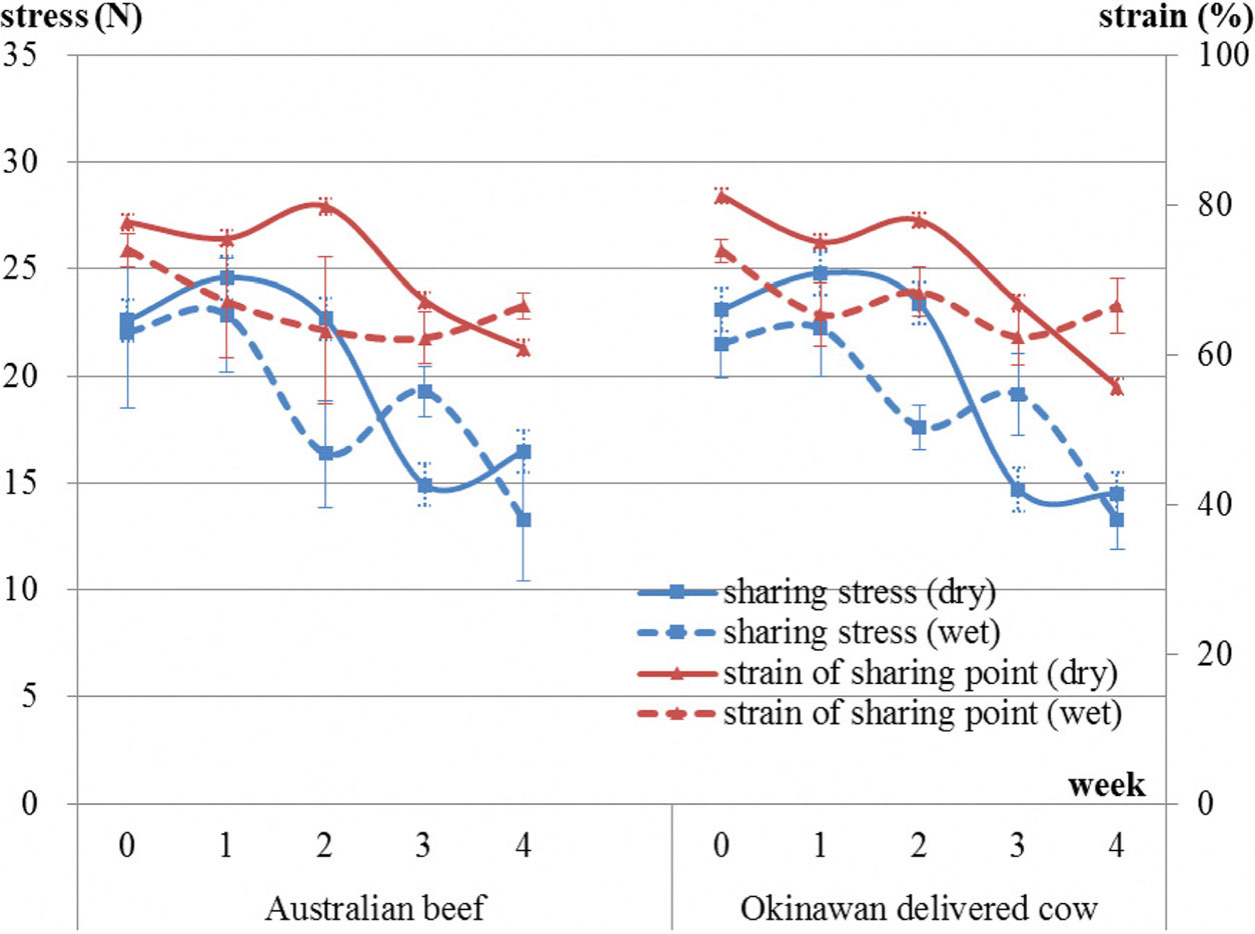
Changes in shearing stress and strain of shearing point in both types of beef during both dry- and wet-aging processes

There were approximately decreasing trends in breaking stress [dry aging of Australian beef: *P* < 0.05, wet aging of Australian beef: *P* = 0.09, dry aging of Okinawan delivered cow beef: (*P* < 0.05), and wet aging of Okinawan delivered cow beef: *P* < 0.05] (Fig. 5). In addition, there were approximately decreasing trends in sharing stress [dry aging of Australian beef: *P* = 0.1 and wet aging of Australian beef: *P* < 0.05 and dry aging of Okinawan delivered cow beef: *P* = 0.086 and wet aging of Okinawan delivered cow beef: *P* = 0.136] (Fig. 6). Probability values for the strain regarding breaking point and shearing point were also determined (breaking point in Australian beef during dry- and wet-aging processes: *P* = 0.52 and *P* = 0.7, respectively; shearing point in Australian beef during dry- and wet-aging processes: *P* = 0.8 and *P* = 0.1, respectively; breaking point in Okinawan delivered cow beef during dry- and wet-aging processes: *P* = 0.054 and *P* = 0.059, respectively; and shearing point in Okinawan delivered cow beef during dry- and wet-aging processes: *P* = 0.055 and *P* = 0.658, respectively). Australian beef has a decreasing trend about only shearing point during wet-aging process, contrary to the case for Okinawan delivered cow beef (Figs. 5 and 6).

Upon comparison of drip loss, cooking loss, and the sum of these between beef types and between aging methods (Table 3), there were significant differences (*P* < 0.01) between beef types for all these variables, which was also the case (P < 0.01) between the aging methods. In the comparison of each amino acid group between beef types and between aging methods (Table 4), there were significant differences between beef types regarding function (*P* < 0.05), sweetness (*P* < 0.01), and total (*P* < 0.05). However, there was no significant difference between the aging methods for all studied variables even for all amino acids (data of amino acids not shown). Comparison of rheological properties between beef types and between aging methods showed there was a significant difference between beef types regarding breaking stress alone (Table 5).

**Table 3:** Comparison of drip loss, cooking loss, and their sum between beef types and between aging methods

	Beef type		Aging method		SEM†	*P* values in ANOVA‡	
	Australian	Okinawan	wet aging	dry aging		Beef type	Aging method
Drip loss	4.7	2.2	4.1	2.8	0.1	**	**
Cooking loss	32.3	27.2	30.7	28.8	0.3	**	**
Sum	37.0	29.4	34.8	31.6	0.4	**	**

Values are least-square means *(n* = 3). †Pooled standard error of the mean. ‡Asterisks indicate **P < 0.01 in analysis of variance

**Table 4:** Comparison of levels of each amino acid group between beef types and between aging methods

	Beef type		Aging method		SEM†	*P* values in ANOVA‡	
	Australian	Okinawan	wet aging	dry aging		Beef type	Aging method
Function	230.3	185.5	204.4	211.4	3.3	*	ns
Sweet	61	42.9	52.7	51.2	2.5	**	ns
Savory	97.4	111.2	112.7	95.9	10.6	ns	ns
Umami	122.3	120.7	120.3	122.7	3.7	ns	ns
Proteinogen ic amino acids	295.4	289.6	300.8	284.2	16.1	ns	ns
Total amino acids	525.7	475	505.2	495.6	14.9	*	ns

Values are least-square means *(n* = 3). †Pooled standard error of the mean. ‡Asterisks indicate **P < 0.01 and *P < 0.05 in analysis of variance

**Table 5:** Comparison of rheological properties between beef types and between aging methods

	Beef type		Aging method		SEM†	*P* values in ANOVA‡	
	Australian	Okinawan	wet aging	dry aging		Beef type	Aging method
Breaking stress (Pa)	1.3E+06	1.1E+06	1.2E+06	1.3E+06	6.9E+04	*	ns
Strain of breaking point (%)	68.4	64.7	66.8	66.3	1.6	ns	ns
Sharing stress (N)	20.6	19.5	19.7	20.5	9.6E+05	ns	ns
Strain of sharing point (%)	74.5	69.4	72.8	71.1	2	ns	ns

Values are least-square means *(n* = 3). †Pooled standard error of the mean. ‡Asterisks indicate *P < 0.05 in analysis of variance

## Discussion

The present study demonstrated the effects of both dry- and wet-aging processes on Okinawan delivered cow beef and Australian beef. Productive loss increased as the number of days of dry aging increased. However, there was no productive loss, such as moisture and trim losses, during the wet-aging process. This shows that the price of dry-aged beef must be higher than that of wet-aged beef to compensate for this loss. In our study, productive loss from both beef types increased to > 30% under conditions of approximately 80% relative humidity for 4 weeks. Many of the compounds responsible for flavor are concentrated by the dry-aging process, according to USMEF [7]. In other words, the distinguishing effect of the dry-aging process on beef is that it concentrates the flavor [3, 7, 11]. Therefore, it is considered that amino acids in beef are also concentrated during the dry-aging process. On the contrary, as described in Introduction, wet-aged steaks had significantly higher sour flavor than dry-aged steaks [11] and wet-aged beef had significantly greater percentages of acids than dry-aged beef [4]. Free amino acid is one kind of acids and possibly contributes to sour flavor of wet-aged beef. In our study, the change in levels of amino acids during the dry-aging process was compared with that during the wet-aging process. The meat from Okinawan delivered cow beef seemed to have a higher increase in proteinogenic amino acids during the dry-aging process than during the wet-aging process, particularly in the middle of the aging process, contrary to the case for Australian beef. However, finally, 4 weeks after aging, these increases during both aging processes were about the same in each type of beef. On ANNOVA, there was no significant difference between aging methods for all studied variables related to free amino acids.

It is known that there are many steps in the degradation of proteins to produce free amino acids. The way to produce free proteinogenic amino acids assumes to be from short peptide not directly from protein. Therefore, it is difficult to elucidate the difference in the underlying mechanism of amino acid production between dry- and wet-aging processes by molecular biological techniques. As such, in the current study, we cannot draw definitive conclusions that the dry-aging process can concentrate amino acids to a greater extent than the wet-aging process and vice versa.

The results showed that glutamic acid increased stably, which is essential for the umami taste of meat, for beef during both types of aging. Sugioka et al. [9] reported that meat from Japanese brown cattle have high levels of glutamic acid and leucine, which increase stably during the aging process. However, alanine did not increase but fluctuated sharply and non-proteinogenic amino acids also did not increase during the aging process. The results in our study were very similar to these results. In particular, the results in Okinawan delivered cow beef were exactly the same as these.

The trend of a decrease in hardness during the dry-aging process is almost the same as that during the wet-aging process. It is difficult to analyze a non-uniform tissue, such as a piece of meat, using a hardness test because of its scattered value of measurement. At this point, the only assertion that can be safely made is that there were similar decreasing trends in hardness during both aging processes. However, the tender I bit them is the clear difference between both dry- and wet-aged beef. Upon chewing wet-aged beef, its tenderness seemed to remain for longer than that of dry-aged beef, whereas dry-aged beef was much easier to cut with the teeth.

The hardness of dry-aged beef gradually decreases as the number of days of aging increases [10]. In addition, Sitz et al. [8] reported that for both the Prime and Choice comparisons, Warner-Bratzler shear force values did not differ between the dry- and wet-aged steaks [8]. These are similar to the results in our study. From the results of the strain regarding breaking point and shearing point, deformation process of meats in both type beefs while being pressed seem to be totally different.

The positive effects, such as an increase in free amino acids and a decrease in hardness, in dry-aged beef were almost the same as those found in wet-aged beef in our study. In fact, there were no significant differences between the two aging methods for all studied variables, except drip, cooking, and productive losses. On the other hand, some significant differences between the beef types were identified on ANOVA, particularly regarding free amino acids. However, it is difficult to study why these differences occurred because these types of beef have different genetic factors and have undergone different fattening methods and for different periods since slaughter.

The present study shows the results of the dry-aging process under conditions without the inoculation of microorganisms. However, in actual conditions, certain molds can grow on the surface of the meat during the dry-aging process, which is one factor that confers the taste of dry-aged beef [3]. Therefore, further studies are needed in demonstrate the effects of mold on dry-aged beef.

## Conclusion

There was no significant difference between dry- and wet-aging methods for all studied variables related to free amino acids or hardness in this study.
